# Association of Cardiovascular Disease With Premature Mortality in the United States

**DOI:** 10.1001/jamacardio.2019.3891

**Published:** 2019-10-16

**Authors:** Yingxi Chen, Neal D. Freedman, Paul S. Albert, Rachel R. Huxley, Meredith S. Shiels, Diana R. Withrow, Susan Spillane, Tiffany M. Powell-Wiley, Amy Berrington de González

**Affiliations:** 1 Division of Cancer Epidemiology and Genetics, National Cancer Institute, Rockville, Maryland; 2 College of Science, Health and Engineering, La Trobe University, Melbourne, Australia; 3 Social Determinants of Obesity and Cardiovascular Risk Laboratory, National Heart, Lung, and Blood Institute, National Institutes of Health, Bethesda, Maryland

## Abstract

**Importance:**

Cardiovascular disease (CVD) is a leading cause of morbidity and mortality in the United States. Despite substantial declines in CVD mortality rates during past decades, progress against cardiovascular deaths in midlife has stagnated, with rates increased in some US racial/ethnic groups.

**Objective:**

To examine the trends in premature (ages 25-64 years) mortality from CVD from 2000 to 2015 by demographics and county-level factors, including education, rurality, and the prevalence of smoking, obesity, and diabetes.

**Design, Setting, and Participants:**

This descriptive study used US national mortality data from the Surveillance, Epidemiology, and End Results data set and included all CVD deaths among individuals ages 25 to 64 years from January 2000 to December 2015. The data analysis began in February 2018.

**Exposures:**

Age, sex, race/ethnicity, and county-level factors.

**Main Outcomes and Measures:**

Age-standardized mortality rates and average annual percent change (AAPC) in rates by age, sex, race/ethnicity, and county-level factors (in quintiles) and relative risks of CVD mortality across quintiles of each county-level factor.

**Results:**

In 2000 to 2015, 2.3 million CVD deaths occurred among individuals age 25 to 64 years in the United States. There were significant declines in CVD mortality for black, Latinx, and Asian and Pacific Islander individuals (AAPC: range, −1.7 to −3.2%), although black people continued to have the highest CVD mortality rates. Mortality rates were second highest for American Indian/Alaskan Native individuals and increased significantly among those aged 25 to 49 years (AAPC: women, 2.1%; men, 1.3%). For white individuals, mortality rates plateaued among women age 25 to 49 years (AAPC, 0.05%). Declines in mortality rates were observed for most major CVD subtypes except for ischemic heart disease, which was stable in white women and increased in American Indian/Alaska Native women, hypertensive heart disease, for which significant increases in rates were observed in most racial/ethnic groups, and endocarditis, for which rates increased in white individuals and American Indian/Alaska Native men. Counties with the highest prevalence of diabetes (quintile 5 vs quintile 1: relative risk range 1.6-1.8 for white individuals and 1.4-1.6 for black individuals) had the most risk of CVD mortality.

**Conclusions and Relevance:**

There have been substantial declines in premature CVD mortality in much of the US population. However, increases in CVD mortality before age 50 years among American Indian/Alaska Native individuals, flattening rates in white people, and overall increases in deaths from hypertensive disease suggest that targeted public health interventions are needed to prevent these premature deaths.

## Key Points

**Question:**

How have the trends in premature mortality from cardiovascular disease (CVD) changed in the United States during 2000 to 2015 by demographics and county-level factors, including education, rurality, and the prevalence of smoking, obesity, and diabetes?

**Findings:**

In this study, CVD mortality rates increased significantly among American Indian/Alaska Native individuals age 25 to 49 years and plateaued among white women in the same age group. County-level diabetes prevalence showed the strongest association with mortality from CVD.

**Meaning:**

Identifying groups/characteristics of counties where rates and trends are substantially high is important for targeted public health interventions.

## Teaser

This descriptive study examines trends in premature mortality from cardiovascular disease from 2000 to 2015 in US individuals age 25 to 64 years.

## Introduction

Cardiovascular diseases (CVDs) are the leading cause of mortality and morbidity in the United States, accounting for more than 900 000 deaths and 14 million disability-adjusted life-years in 2016 alone.^1^ Substantial progress has been made in combating CVD over the past 40 years, with mortality rates falling from 507 in 100 000 in 1980 to 253 in 100 000 in 2014.^2^ However, considerable disparities by race/ethnicity and geographic region persist.^2,3,4^ The American Heart Association has set a strategic goal of reducing deaths from CVD by 20% during 2010 to 2020.^5^ Similar goals have been proposed as part of the Million Hearts Initiative, which aims to prevent a million cardiovascular events during 2017 to 2022.^6^

Nevertheless, recent evidence suggests that progress made against premature mortality from heart disease has stagnated and rates have even increased in some US subgroups over the past 15 years.^7^ However, it remains unclear how trends in the specific type of CVD vary across population subgroups as well as the associated contributions of specific risk factors to CVD mortality. To better characterize 2000 to 2015 trends in rates of CVD premature mortality in the United States, we describe trends and patterns in cardiovascular deaths among people age 25 to 64 years by type of CVD, age group, sex, race/ethnicity, state, and county-level characteristics, including education, rurality, and the prevalence of smoking, obesity, and diabetes. The selection of this group is because (1) CVD death rates continue to decline among individuals 65 years and older in the United States^8^ and (2) the reduction of premature mortality is one of the United Nations Sustainable Development Goals^9^ and CVD mortality in the United States is divergent. A more detailed characterization of trends over time may provide insight into factors associated with increases and decreases in CVD deaths, which ultimately may inform the prioritization of prevention strategies at local and national levels.

## Methods

### Data Sources

Causes of death and demographics were ascertained from national death certificate data from the Surveillance, Epidemiology, and End Results data set, which contains national mortality data from 2000 to 2015. Institutional review board approval and informed consent were waived because the study used publicly available deidentified data. This analysis focused on premature deaths due to CVD, defined based on the *International Statistical Classification of Diseases and Related Health Problems, Tenth Revision (ICD-10)* codes: all CVD (I00-02, I05-09, I10-15, I20-I25, I26-I28, I60-69, I70-79, and I30-51), including ischemic heart disease (I20-25), cerebrovascular disease (I60-69), rheumatic heart disease (I00-02 and I05-09), hypertensive heart disease (I10-15), peripheral arterial disease (I70-79), heart failure (I42, I43, I50), cardiac arrest (I46), arrhythmia (I44-49), and endocarditis (I33 and I38). Age-, sex-, and race/ethnicity–specific data were ascertained from the US Census intercensal populations.

### County Attributes

We assessed the contribution of 5 county-level risk factors to CVD premature mortality: population with a bachelor degree (%), rurality, prevalence of smoking, diabetes, and obesity (defined as a body mass index [calculated as weight in kilograms divided by height in meters squared] of ≥30). We selected these 5 county-level factors based on their association with CVD mortality. Cardiovascular deaths vary by socioeconomic status. Therefore, we included county-level bachelor degree and rurality as proxies for socioeconomic status. We also included smoking, obesity, and diabetes prevalence because they are common risk factors for CVD and mortality.

Because county-level socioeconomic status (SES) characteristics from 2011 to 2015 have been reported to be most relevant to current mortality rates,^10^ we ascertained the percentage of individuals with a bachelor degree (based on a population 25 years or older) from the 2011 to 2015 Census American Community Survey.^11^ The rurality of counties was defined based on the 2013 Rural-Urban Continuum codes that were developed by the US Department of Agriculture.^12^ Data on county-level smoking prevalence during 2008 to 2010 for 18 years or longer were ascertained from the Behavioral Risk Factor Surveillance System and the National Health Interview Survey.^13,14^ County-level obesity and diabetes prevalence for a population 20 years or older were estimated based on the Institute for Health Metrics and Evaluation.^15^

### Statistical Analysis

We estimated age-standardized mortality rates (ASRs) overall and stratified by calendar period (2000–2003 and 2012–2015), age group (25-49 years and 50-64 years), sex, and race/ethnicity (non-Hispanic white [ie, white], non-Hispanic black [ie, black], Asian and Pacific Islander, American Indian and Alaska Native, and Latinx) using the standard 2000 US population in 5-year age groups as the reference. The selection of these 2 age groups was based on published data examining premature all-cause mortality in the United States by county using the US death certificate data.^10^ We restricted estimates for American Indian/Alaska Native individuals to contract health services delivery areas to reduce racial/ethnic misclassification.^16^ We also estimated the average annual percentage change (AAPC) in rates during 2000 to 2015 by age group, sex, state, and race/ethnicity stratified by CVD types. Analyses were conducted using SEER*Stat software (National Cancer Institute).

We then estimated age-standardized CVD mortality rates during 2012 to 2015 among white, black, and Latinx individuals by each county-level factor. We categorized counties by the following county-level factors: (1) percentage with a bachelor degree (1%-20%, 21%-26%, 27%-30%, 31%-37%, and 38%-79%), (2) rurality (metropolitan areas with ≥1 million people, metropolitan areas with 250 000 to <1 million people, metropolitan areas of <250 000 people, urban areas with ≥20 000 people, urban areas with 2500 to <20 000 people, and completely rural populations with <2500 people), (3) smoking prevalence (6%-20%, 21%-23%, 24%-26%, 27%-29%, and 30%-43%), (4) obesity prevalence (18%-34%, 35%-36%, 37%-38%, 39%-40%, and 41%-53%), and (5) diabetes prevalence (5%-8.5%, 8.6%-9.7%, 9.8%-10.6%, 10.7%-11%, and 12%-21%). Missing values were categorized to a separate group. Because we aggregated counties, we included all counties regardless of population size and number of deaths. We also estimated the annual percentage change in mortality rates during 2000 to 2015 by the level of each county-level factor.

We then conducted a multivariate quasi-Poisson regression, adjusting for age (5-year age group) and 5 county-level factors to examine the associations of these risk factors with recent CVD mortality rates (rates in 2012-2015), including all CVD, ischemic heart disease, and hypertensive heart disease. Because the total number of events was too sparse for certain races/ethnicities after stratifying by county-level risk factors, we restricted the regression analyses to white and black individuals. Regression analyses were conducted using the R program, version 3.4.2 (R Foundation) and statistical significance was set at *P *< .05.

## Results

### CVD Mortality

During 2000 to 2015, more than 2.3 million CVD deaths occurred among individuals aged 25 to 64 years in the United States. Among women, the ASR declined by 20% from 60 in 100 000 during 2000 to 2003 to 48 in 100 000 during 2012 to 2015 (AAPC, −1.9%). Similarly, among men, the ASR declined by 20% from 130 in 100 000 during 2000 to 2003 to 104 in 100 000 during 2012 to 2015 (AAPC, −1.8%).

There was substantial variation in total CVD mortality rates and trends by racial/ethnic and age groups. Among women, black individuals had the highest ASR during 2012 to 2015 (101/100 000), followed by American Indian/Alaska Native (74/100 000), white (44/100 000), and Latinx individuals (28/100 000). Asian Pacific Islander individuals had the lowest ASR (19/100 000). Similarly, in men, ASR during 2012 to 2015 were highest among black individuals (190/100 000), followed by American Indian/Alaska Native (161/100 000), white (100/100 000), Latinx (69/100 000), and Asian Pacific Islander individuals (55/100 000) (Table).

**Table.** Trends and Age-Standardized Premature Mortality (Age 25 to 64 Years) Rates Because of All Cardiovascular Disease in the United States During 2000 to 2003 Compared With 2012 to 2015^a^

Race/Ethnicity	Age 25-49 y			Age 50-64 y			Overall		
	2000-2003 per 100 000	2012-2015 per 100 000	Average Annual % Change	2000-2003 per 100 000	2012-2015 per 100 000	Average Annual % Change	2000-2003 per 100 000	2012-2015 per 100 000	Average Annual % Change
**Women**									
White	19	19	0.05	129	104	−1.8	51	44	−1.3
Black	62	46	−2.5	341	237	−3.0	143	101	−2.9
Latinx	14	11	−2.0	108	72	−3.3	41	28	−3.0
Asian Pacific Islander	10	7	−2.0	75	49	−3.6	28	19	−3.2
American Indian/Alaska Native	31	40	2.1	185	158	−1.2	75	74	−0.1
Overall	24	21	−1.1	149	114	−2.2	60	48	−1.9
**Men**									
White	47	41	−1.1	305	247	−1.7	121	100	−1.9
Black	102	85	−1.6	619	450	−2.7	251	190	−2.4
Latinx	31	26	−1.6	241	176	−2.6	92	69	−2.3
Asian Pacific Islander	26	23	−0.9	168	133	−2.0	67	55	−1.7
American Indian/Alaska Native	67	77	1.3	381	368	−0.2	157	161	0.3
Overall	51	43	−1.4	327	258	−2.0	130	105	−1.8

^a^ Rates per 100 000.

Black individuals showed statistically significant declines in ASR during 2000 to 2015 for women and men (AAPC, −2.9% in women; −2.4% in men), along with Latinx (−3.0% in women; −2.3% in men), Asian Pacific Islander (−3.2% in women; −1.7% in men), and white individuals (−1.3% in women; -1.9% in men). In contrast, ASR increased significantly among American Indian/Alaska Native individuals age 25 to 49 years (AAPC: women, 2.1%; men, 1.3%), and ASR among white women aged 25 to 49 years plateaued (AAPC, 0.05%) (Table).

**Figure 1.**
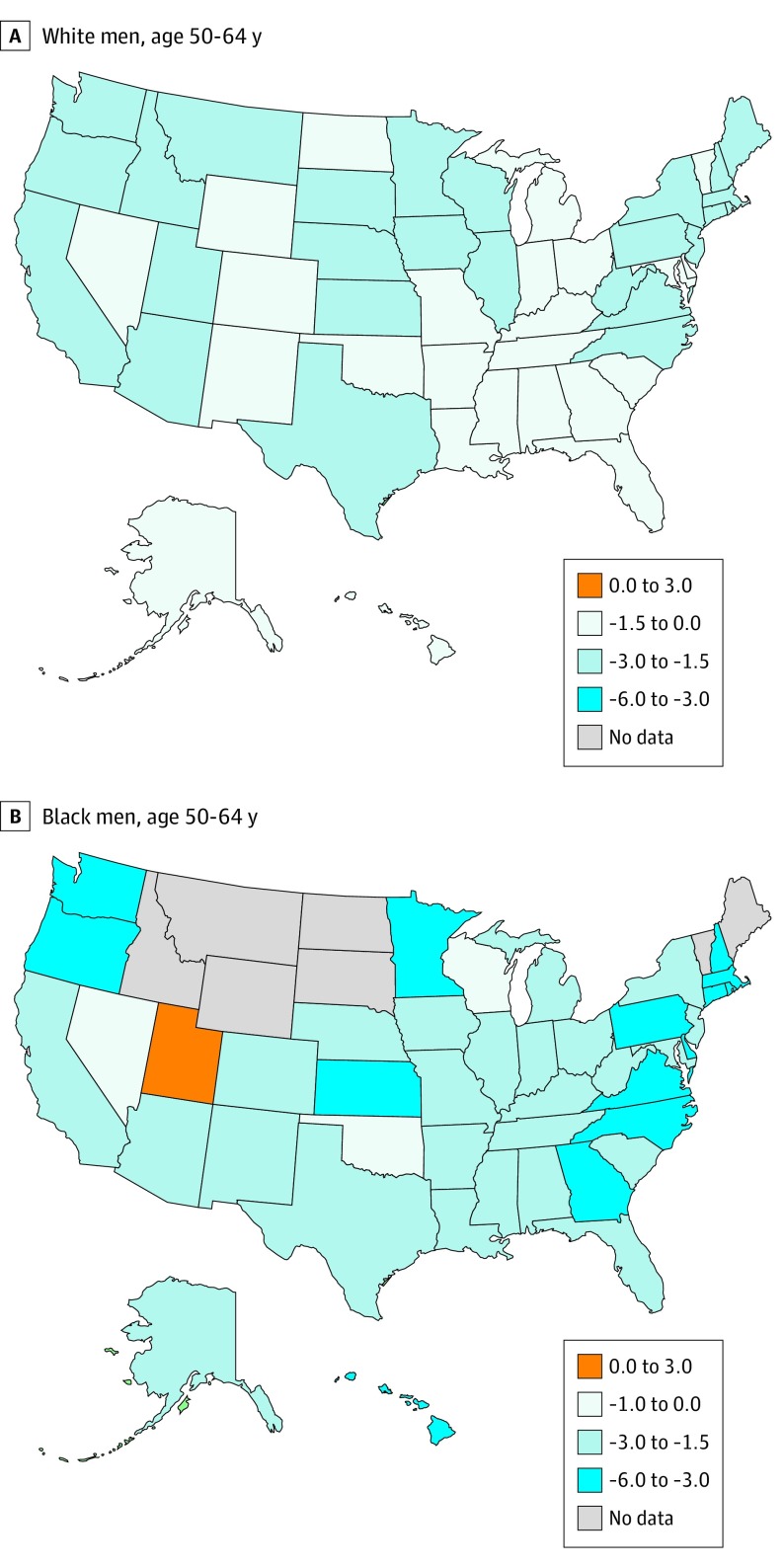
Average Annual Percentage Change of Cardiovascular Disease Mortality Rates Among White and Black Men Aged 50 to 64 Years by State, 2000 to 2015

The analysis of state-specific trends was limited to black and white individuals because of sparse data in other racial/ethnic groups. During 2000 to 2015, most groups of black and white individuals showed significant declines in CVD mortality, particularly among men aged 50 to 64 years; whereas white men had significant declines in all states, black men had more pronounced declines in most states, except Utah (AAPC = 2.8%) (Figure 1). However, compared with other groups, white women aged 25 to 49 years showed considerable variations in trends, with AAPCs ranging from −3.3% in Oregon to 2.3% in Alabama (eFigure 1 in the Supplement).

Ischemic heart disease (54% of all CVD premature deaths), cerebrovascular disease (13%), and hypertensive heart disease (10%) contributed to the largest fraction of CVD premature deaths from 2000 to 2015. Declines in ASR due to ischemic heart disease were the primary contributor to the decline in overall CVD mortality, with decreases in most age and racial/ethnic groups (AAPC: range, −4.7 to −0.5%). Mortality declines in ASR were also observed for cerebrovascular disease (−4.5% to −0.4%), peripheral arterial disease (−5.5% to −0.1%), and rheumatic heart disease (−6.6% to −0.3%) (eTable 1 in the Supplement). In contrast, ASRs of ischemic heart disease increased among American Indian/Alaska Native women aged 25 to 49 years (AAPC: 1.7%) and were stagnant for white women aged 25 to 49 years (eTable 1 in the Supplement).

**Figure 2.**
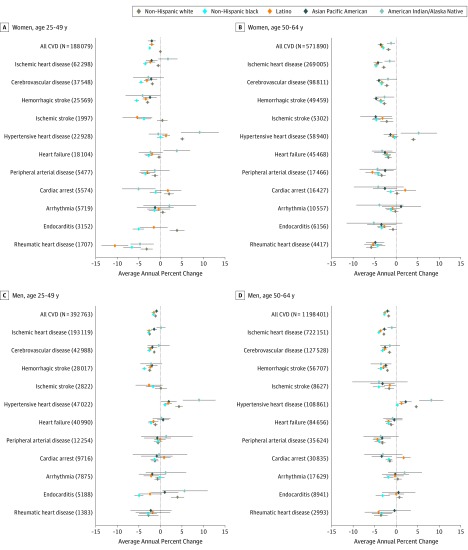
Cardiovascular Disease (CVD) Mortality Average Annual Percentage Change Stratified by Age Group, Sex, and Race/Ethnicity Across Types of Disease, 2000 to 2015

During 2000 to 2015, ASR from hypertensive heart disease, which was the third most common cause of CVD deaths in 2012 to 2015, increased in most racial/ethnic groups (Figure 2). Compared with other groups, white women aged 25 to 49 years had the highest increase in hypertensive heart disease mortality. An additional analysis of trends showed increases in ASR of hypertensive heart disease in most states (AAPC, from 0.4% in South Carolina to 19.7% in South Dakota), except Washington, DC (−2.5%) and Vermont (−1.6%) (eFigure 2 in the Supplement). The ASR of endocarditis increased significantly in white individuals aged 25 to 49 years (AAPC: women, 3.9%; men, 3.7%) and American Indian/Alaska Native men (5.6%) (Figure 2).

### CVD Mortality by County-Level Factors

**Figure 3.**
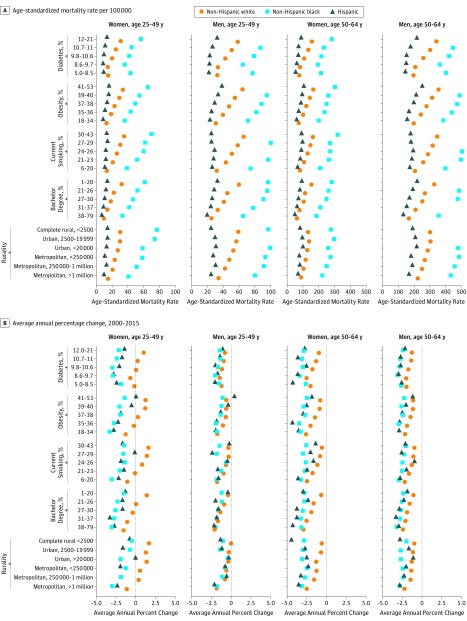
County-Level Mortality Rates A, Age-standardized all cardiovascular disease (CVD) mortality rates, 2012 to 2015. B, Average annual percentage change of all CVD mortality rates, 2000 to 2015.

**Figure 4.**
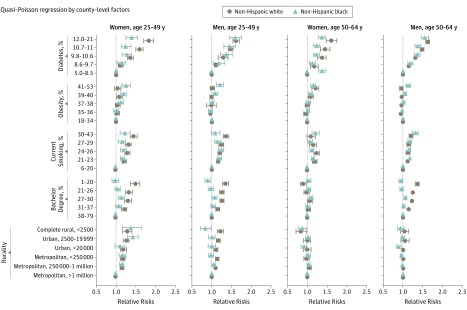
Multivariate Quasi-Poisson Regression by County-Level Risk Factors, 2012 to 2015

Recent CVD premature mortality rates (2012–2015) varied substantially by county-level risk factors. The ASRs were consistently higher in counties with lower socioeconomic factors (ie, lower education levels and rural areas). The ASRs were also generally higher in counties with a high prevalence of diabetes, obesity, and smoking. These increases followed a gradient across quintiles of county-level factors and were more pronounced in white than black and Latinx individuals (Figure 3A). During 2000 to 2015, ASR among black and Latinx individuals declined over time across counties regardless of county-level factors. In contrast, ASR among white women aged 25 to 49 years predominantly increased among counties with a lower percentage of bachelor degrees, and a higher prevalence of diabetes, obesity, and smoking and among more rural counties (Figure 3B). A multivariate quasi-Poisson regression confirmed associations between county-level factors and the risk of CVD mortality, in which county-level diabetes prevalence showed the strongest effect (Figure 4). The associations were more pronounced in white individuals aged 25 to 49 years than black (Figure 4). For example, for women aged 25 to 49 years, compared with counties with a diabetes prevalence of 5% to 8.5%, the risk of CVD mortality was 1.4 times higher among white individuals and 1.3 times higher among black individuals who lived in counties with a diabetes prevalence of 9.8% to 10.6%; the risk of CVD mortality was 1.8 times higher among white individuals and 1.4 times higher among black individuals in counties with a diabetes prevalence of 12% to 21% (Figure 4 and eTable 2 in the Supplement).

As mortality rates of hypertensive heart disease increased significantly across most age, sex, and racial/ethnic groups, we further examined rates and trends by county-level factors. Age-standardized mortality rates of hypertensive heart disease were generally higher in counties with a high prevalence of diabetes and obesity, but the associations between county-level smoking prevalence and education levels and mortality rates were less pronounced (eFigure 3 in the Supplement). Increases occurred across many types of counties but generally followed a gradient across quintiles of county-level factors, although these patterns were less clear among black and Latinx individuals (eFigure 3 in the Supplement). The multivariate quasi-Poisson regression showed a predominant association between county-level diabetes prevalence and hypertensive heart disease mortality (eFigure 3 and eTable 3 in the Supplement).

## Discussion

During 2000 to 2015, we observed significant declines in CVD premature mortality rates for black, Latinx, and Asian and Pacific Islander individuals but significant increases in rates among American Indians/Alaska Native individuals age 25 to 49 years; rates plateaued among white women aged 25 to 49 years. These increases were driven by a lack of progress against mortality from ischemic heart disease and increases in mortality from hypertensive heart disease and endocarditis.

The American Heart Association has set a strategic goal for reducing deaths from CVD by 20% from 2010 to 2020.^5^ While there was an overall 28% decrease in CVD mortality rates in the United States during 2003 to 2015,^17^ reflecting the progress made in prevention and advances in cardiovascular disease treatment, equivalent declines have not been observed across age groups.^18^ Improvements in mortality also vary by race/ethnicity. A recent US Centers for Disease Control and Prevention report examining mortality trends found that heart disease death rates increased for white people aged 45 to 64 years during 2009 to 2017 and for black individuals aged 45 to 64 years during 2011 to 2017 despite an overall of 22% of decline in rates from 1999 to 2011.^19^ In this study, we compared premature mortality due to all CVD during 2012 to 2015 with rates from 2000 to 2003. While we observed declines in CVD mortality, we found stagnation in ischemic heart disease mortality among young white women and significant increases in mortality from hypertensive heart disease across most races/ethnicities. This highlights the importance of understanding the diversity and disparities in CVD mortality by risk factors among different groups.

Disparities in CVD mortality by SES have been well established in the United States.^20^ Important risk factors, including smoking, obesity, and diabetes, are more prevalent among lower-SES counties compared with higher-SES counties.^21^ As observed previously,^2,7^ we found substantial differences in mortality rates by age, sex, and racial/ethnic groups across different types of counties. For example, CVD mortality rates were generally lower in high-SES counties and more urban areas. These findings highlight potential opportunities among states to target public health resources for primary and secondary CVD prevention toward counties based on SES status and rurality.^22^

In contrast to the trends for overall CVD mortality, we found marked increases in premature mortality associated with hypertensive heart disease across most age and racial/ethnic groups. This increase occurred across the country, although gradients by county characteristics were noted. Previous data suggest that only half of US individuals with hypertension had their condition effectively controlled.^23^ Consistent with our data, the proportion of US mortality rates attributable to hypertension increased by 10.5% from 2005 to 2015 and the absolute number of deaths attributable to hypertension rose by 37.5%.^24^ Hypertension is common in patients with diabetes^25^ and patients with diabetes and hypertension experience more severe cardiovascular outcomes than patients with either condition alone.^25^ We found that compared with other risk factors, diabetes prevalence had the strongest association with premature mortality from hypertensive heart disease.

Without widespread intervention, continued increases in diabetes prevalence in the United States will likely exacerbate the adverse trends observed here. Coupled with this, increases in premature mortality associated with hypertensive heart disease are most concerning given the change in the hypertension definition in the 2017 clinical guidelines.^26^ The new guidelines markedly reset the diagnosis and treatment of hypertension, which potentially results in more individuals receiving a hypertension diagnosis.^27^ Public health policies to mitigate these trends in CVD mortality will need to focus on diagnosis, treatment, and control of hypertension in younger populations, particularly in lower-resourced, underserved communities. In addition, continuously monitoring mortality from hypertensive heart disease is important to evaluate the effect of changes in the clinical guideline and how this may shape the CVD mortality rates overall.

The significant increase in premature mortality that is associated with endocarditis among young white individuals and young American Indian and Alaska Native men is also worrisome, despite relatively few deaths. Coincident with the marked rise in deaths from opioid use, increase in endocarditis among certain groups may be associated with the opioid epidemic. Although this study could not directly address this hypothesis, a disproportionate increase in deaths from endocarditis has been found among young white individuals who inject drugs.^28^ In 2016, approximately 1 million people used heroin in the United States, which was 2-fold higher than the total number of heroin users in the period over 2002 to 2013.^29^ Consequently, many of the population were at risk of bacteremia from contaminated needles and subsequent endocarditis. Future studies investigating the association of injection drug use with trends in endocarditis are needed.

### Strengths and Limitations

A strength of this study was the stratification of the analysis by age groups, allowing us to identify trends that would be missed across the full age range by age standardization. Another strength of this study was the estimation and comparison of county-level CVD risk factors. County-level estimates enable the identification of local trends and patterns of CVD mortality that are associated with important risk factors. However, as macro-level estimates, county-level risk factors do not represent the factors of specific individuals who died of CVD nor age- and sex-specific variations within each county. Additionally, county-level risk factor estimates were restricted to white, black, and Latinx individuals as there were fewer deaths occurring among other groups, which precluded systematic examination by county.

Another limitation of this study is the use of death certificate data. Racial/ethnic misclassification is a main concern, as race/ethnicity data on death certificates are typically recorded by funeral directors, whereas self-reported information on the census is used to calculate the denominators in this study. Nevertheless, race/ethnicity recording on death certificates has been reported as highly accurate for white and black individuals, and misclassification in Latinx and Asian and Pacific Islander individuals is minor.^30^ To reduce misclassification in American Indian and Alaska Native individuals, we restricted the analysis to contract health services delivery areas.^16^ There are also concerns about the possible misclassification of CVD and changes in coding over time. Use of these data for CVD in aggregate has been validated and reduces the potential for misclassification.^31,32^ However, we should be cautious regarding examining trends in hypertensive heart disease and other types. For example, with increasing emphasis on blood pressure control, deaths from heart failure, coronary artery disease, and stroke may be misclassified as hypertensive heart disease. This misclassification may overestimate the effect of hypertensive heart disease, but it may also underestimate the effect of other heart diseases. Despite this, the use of death certificate data is the most feasible current method to conduct this type of analysis on the US population, as opposed to community-based cohorts in which the findings would have limited generalizability. Additionally, as CVD in aggregate includes several diseases with distinct etiologies, it is important to explore the contributions of individual types of CVD to the overall picture over time.

Identifying groups with increasing CVD mortality rates is important to guide targeted prevention efforts. Recent work identifies important areas of research to better understand the high rates of CVD mortality among American Indian and Alaska Native populations, including work to elucidate the association of adverse childhood experiences and other psychosocial factors with CVD in these communities.^33^ Future interventions, policies, and systems approaches to promote healthy behaviors and reduce CVD risk are needed among American Indian and Alaska Native youth. For younger women at risk for CVD, including young white women, screening for CVD risk factors must be considered across medical specialties that regularly care for women.^34^ Tailored interventions to promote cardiovascular health may most easily reach women outside of the clinical setting by leveraging social media and mobile health technology.^35^

## Conclusions

Despite the marked progress that has been made in preventing cardiovascular deaths in the United States, substantial increases in CVD mortality rates have occurred among American Indians and Alaska Native individuals aged 25 to 49 years and rates have plateaued among young white women, reflecting a lack of progress against mortality from ischemic heart disease and increases in mortality rates from hypertensive heart disease and endocarditis in these groups. Mortality from hypertensive heart disease is a growing concern that affects most groups. Counties with a high prevalence of obesity, diabetes, and smoking have continued to experience higher rates of CVD mortality and should be targeted for intervention. Although CVD risk factors are modifiable, sustained efforts at such interventions are needed. Without rapid and sustained progress against cardiovascular risk factors, public health goals to further reduce the burden of cardiovascular disease mortality in the United States are unlikely to be met.

## Notes

Supplement. eFigure 1. Average annual percent change of CVD mortality rates by states, stratified by race/ethnicity, sex, and age groups, 2000-15 eFigure 2. Average annual percent change of mortality rates due to hypertensive heart disease by states, 2000-15 eFigure 3. County-level (A) age-standardized hypertensive heart disease mortality rates, 2012-2015; (B) average annual percent change of hypertensive heart disease mortality rates, 2000-2015, and (C) multivariate quasi-Poisson regression by county-level risk factors 2012-15 eTable 1. Trends and age-standardized premature mortality (age 25-64 years) rates due to specific cardiovascular disease in the US, 2000-03 compared to 2012-15, rates per 100,000 eTable 2. Relative risk and 95% confidence intervals for county-level CVD premature mortality by country-level risk factors, adjusting for age and all five county-level risk factors, 2012-15 eTable 3. Relative risk and 95% confidence intervals (CI) for county-level premature mortality due to hypertensive heart disease by county-level risk factors, adjusting for age and all five county-level risk factors, 2012-15

## References

[1] Roth GA, Johnson CO, Abate KH, ; Global Burden of Cardiovascular Diseases Collaboration The burden of cardiovascular diseases among US states, 1990-2016. JAMA Cardiol. 2018;3(5):375-389. doi:10.1001/jamacardio.2018.0385 29641820 PMC6145754

[2] Roth GA, Dwyer-Lindgren L, Bertozzi-Villa A, Trends and patterns of geographic variation in cardiovascular mortality among US counties, 1980-2014. JAMA. 2017;317(19):1976-1992. doi:10.1001/jama.2017.4150 28510678 PMC5598768

[3] Patel SA, Winkel M, Ali MK, Narayan KM, Mehta NK Cardiovascular mortality associated with 5 leading risk factors: national and state preventable fractions estimated from survey data. Ann Intern Med. 2015;163(4):245-253. doi:10.7326/M14-1753 26121190

[4] Singh GK, Siahpush M Increasing inequalities in all-cause and cardiovascular mortality among US adults aged 25-64 years by area socioeconomic status, 1969-1998. Int J Epidemiol. 2002;31(3):600-613. doi:10.1093/ije/31.3.600 12055162

[5] Lloyd-Jones DM, Hong Y, Labarthe D, ; American Heart Association Strategic Planning Task Force and Statistics Committee Defining and setting national goals for cardiovascular health promotion and disease reduction: the American Heart Association’s strategic impact goal through 2020 and beyond. Circulation. 2010;121(4):586-613. doi:10.1161/CIRCULATIONAHA.109.192703 20089546

[6] Wright JS, Wall HK, Ritchey MD Million hearts 2022: Small steps are needed for cardiovascular disease prevention. JAMA. 2018;320(18):1857-1858. doi:10.1001/jama.2018.13326 30193304 PMC8422805

[7] Sidney S, Quesenberry CP Jr, Jaffe MG, Recent trends in cardiovascular mortality in the United States and public health goals. JAMA Cardiol. 2016;1(5):594-599. doi:10.1001/jamacardio.2016.1326 27438477

[8] Centers for Disease Control and Prevention About underlying cause of death, 1999-2017. https://wonder.cdc.gov/ucd-icd10.html#targetText=Underlying%20Cause%20of%20Death%2C%201999%2D2017%20Request&targetText=The%20Underlying%20Cause%20of%20Death,counts%20for%20all%20U.S.%20counties.&targetText=Data%20are%20also%20available%20for,whether%20an%20autopsy%20was%20performed. Accessed March 15, 2018.

[9] Norheim OF, Jha P, Admasu K, Avoiding 40% of the premature deaths in each country, 2010-30: review of national mortality trends to help quantify the UN sustainable development goal for health. Lancet. 2015;385(9964):239-252. doi:10.1016/S0140-6736(14)61591-9 25242039

[10] Shiels MS, Berrington de González A, Best AF, Premature mortality from all causes and drug poisonings in the USA according to socioeconomic status and rurality: an analysis of death certificate data by county from 2000-15. Lancet Public Health. 2019;4(2):e97-e106. doi:10.1016/S2468-2667(18)30208-1 30655229 PMC6392082

[11] American Community Survey Office 2015 ACS 1-year and 2011-2015 ACS 5-year data releases. https://www.census.gov/acs/www/data/data-tables-and-tools/data-profiles/2015/. Accessed February 5, 2018.

[12] United States Department of Agriculture 2013 rural-urban continuum codes documentation. https://www.ers.usda.gov/data-products/rural-urban-continuum-codes/. Accessed February 15, 2018.

[13] National Cancer Institute (U.S.) Data sources for the model-based small area estimates of cancer risk factors and screening behaviors. https://sae.cancer.gov/nhis-brfss/. Accessed January 20, 2018.

[14] National Cancer Institute (U.S.) Methodology for the model-based small area estimates of cancer risk factors & screening behaviors. https://sae.cancer.gov/nhis-brfss/methodology.html. Accessed January 20, 2018.

[15] Institute for Health Metrics and Evaluation http://www.healthdata.org/data-tools. March 19, 2018.

[16] Jim MA, Arias E, Seneca DS, Racial misclassification of American Indians and Alaska Natives by Indian Health Service contract health service delivery area. Am J Public Health. 2014;104(suppl 3):S295-S302. doi:10.2105/AJPH.2014.301933 24754617 PMC4035863

[17] Hastings KG, Boothroyd DB, Kapphahn K, Socioeconomic differences in the epidemiologic transition from heart disease to cancer as the leading cause of death in the United States, 2003 to 2015:an observational study. Ann Intern Med. 2018;169(12):836-844. doi:10.7326/M17-0796 30422275

[18] Ford ES, Ajani UA, Croft JB, Explaining the decrease in U.S. deaths from coronary disease, 1980-2000. N Engl J Med. 2007;356(23):2388-2398. doi:10.1056/NEJMsa053935 17554120

[19] Curtin SC Trends in cancer and heart disease death rates among adults aged 45–64: United States, 1999–2017. Natl Vital Stat Rep. 2019;68(5):1. https://www.cdc.gov/nchs/data/nvsr/nvsr68/nvsr68_05-508.pdf. Accessed June 17, 2019. 32501204

[20] Fretz A, Schneider AL, McEvoy JW, The association of socioeconomic status with subclinical myocardial damage, incident cardiovascular events, and mortality in the ARIC study. Am J Epidemiol. 2016;183(5):452-461. doi:10.1093/aje/kwv253 26861239 PMC4772435

[21] Egen O, Beatty K, Blackley DJ, Brown K, Wykoff R Health and social conditions of the poorest versus wealthiest counties in the United States. Am J Public Health. 2017;107(1):130-135. doi:10.2105/AJPH.2016.303515 27854531 PMC5308159

[22] Vaughan AS, Ritchey MD, Hannan J, Kramer MR, Casper M Widespread recent increases in county-level heart disease mortality across age groups. Ann Epidemiol. 2017;27(12):796-800. doi:10.1016/j.annepidem.2017.10.012 29122432 PMC5733620

[23] Olives C, Myerson R, Mokdad AH, Murray CJ, Lim SS Prevalence, awareness, treatment, and control of hypertension in United States counties, 2001-2009. PLoS One. 2013;8(4):e60308. doi:10.1371/journal.pone.0060308 23577099 PMC3618269

[24] Benjamin EJ, Virani SS, Callaway CW, ; American Heart Association Council on Epidemiology and Prevention Statistics Committee and Stroke Statistics Subcommittee Heart Disease and Stroke Statistics-2018 update: a report from the American Heart Association. Circulation. 2018;137(12):e67-e492. doi:10.1161/CIR.0000000000000558 29386200

[25] Tsimihodimos V, Gonzalez-Villalpando C, Meigs JB, Ferrannini E Hypertension and diabetes mellitus: coprediction and time trajectories. Hypertension. 2018;71(3):422-428. doi:10.1161/HYPERTENSIONAHA.117.10546 29335249 PMC5877818

[26] Yancy CW, Fonarow GC The new hypertension guidelines: compelling population benefit, manageable risk, and time to implement. JAMA Cardiol. 2018;3(7):581-582. doi:10.1001/jamacardio.2018.1264 29800020

[27] Whelton PK, Carey RM, Aronow WS, A guideline for the prevention, detection, evaluation, and management of high blood pressure in adults: executive summary: a report of the American College of Cardiology/American Heart Association Task Force on Clinical Practice Guidelines. J Am Coll Cardiol. 2018;71(19):2199-2269. doi:10.1016/j.jacc.2017.11.005 29146535

[28] Njoroge LW, Al-Kindi SG, Koromia GA, ElAmm CA, Oliveira GH Changes in the association of rising infective endocarditis with mortality in people who inject drugs. JAMA Cardiol. 2018;3(8):779-780. doi:10.1001/jamacardio.2018.1602 29926083 PMC6143071

[29] Substance Abuse and Mental Health Services Administration Key substance use and mental health indicators in the United States: results from the 2016 National Survey on Drug Use and Health. https://www.samhsa.gov/data/. Accessed, March 19, 2018.

[30] Arias E, Heron M, Hakes J; National Center for Health Statistics; US Census Bureau The validity of race and Hispanic origin reporting on death certificates in the United States: an update. Vital Health Stat 2. 2016;(172):1-21. 28436642

[31] Ives DG, Samuel P, Psaty BM, Kuller LH Agreement between nosologist and cardiovascular health study review of deaths: implications of coding differences. J Am Geriatr Soc. 2009;57(1):133-139. doi:10.1111/j.1532-5415.2008.02056.x 19016930 PMC2631612

[32] Coady SA, Sorlie PD, Cooper LS, Folsom AR, Rosamond WD, Conwill DE Validation of death certificate diagnosis for coronary heart disease: the Atherosclerosis Risk in Communities (ARIC) Study. J Clin Epidemiol. 2001;54(1):40-50. doi:10.1016/S0895-4356(00)00272-9 11165467

[33] Deen JF, Adams AK, Fretts A, Cardiovascular disease in American Indian and Alaska Native youth: unique Risk factors and areas of scholarly need. J Am Heart Assoc. 2017;6(10):e007576. doi:10.1161/JAHA.117.007576 29066451 PMC5721901

[34] Nabel EG Heart disease prevention in young women: sounding an alarm. Circulation. 2015;132(11):989-991. doi:10.1161/CIRCULATIONAHA.115.018352 26302760

[35] Burke LE, Ma J, Azar KM, ; American Heart Association Publications Committee of the Council on Epidemiology and Prevention, Behavior Change Committee of the Council on Cardiometabolic Health, Council on Cardiovascular and Stroke Nursing, Council on Functional Genomics and Translational Biology, Council on Quality of Care and Outcomes Research, and Stroke Council Current science on consumer use of mobile health for cardiovascular disease prevention: a scientific statement from the American Heart Association. Circulation. 2015;132(12):1157-1213. doi:10.1161/CIR.0000000000000232 26271892 PMC7313380

