# Supplementary material for: Association of Cardiovascular Disease With Premature Mortality in the United States
Source: JAMA Cardiol. 2019 Oct 16;4(12):1230–8. doi: 10.1001/jamacardio.2019.3891 (PMC6802055; doi:10.1001/jamacardio.2019.3891)
Supplement: Supplement. — eFigure 1. Average annual percent change of CVD mortality rates by states, stratified by race/ethnicity, sex, and age groups, 2000-15 eFigure 2. Average annual percent change of mortality rates due to hypertensive heart disease by states, 2000-15 eFigure 3. County-level (A) age-standardized hypertensive heart disease mortality rates, 2012-2015; (B) average annual percent change of hypertensive heart disease mortality rates, 2000-2015, and (C) multivariate quasi-Poisson regression by county-level risk factors 2012-15 eTable 1. Trends and age-standardized premature mortality (age 25-64 years) rates due to specific cardiovascular disease in the US, 2000-03 compared to 2012-15, rates per 100,000 eTable 2. Relative risk and 95% confidence intervals for county-level CVD premature mortality by country-level risk factors, adjusting for age and all five county-level risk factors, 2012-15 eTable 3. Relative risk and 95% confidence intervals (CI) for county-level premature mortality due to hypertensive heart disease by county-level risk factors, adjusting for age and all five county-level risk factors, 2012-15 [file jamacardiol-4-1230-s001.pdf]

Supplementary Online Content

Chen Y, Freedman ND, Albert PS, et al. Association of cardiovascular disease with premature mortality in the United States. *JAMA Cardiol*. Published online October 16, 2019. doi:10.1001/jamacardio.2019.3891

eFigure 1. Average annual percent change of CVD mortality rates by states, stratified by race/ethnicity, sex, and age groups, 2000-15
eFigure 2. Average annual percent change of mortality rates due to hypertensive heart disease by states, 2000-15
eFigure 3. County-level (A) age-standardized hypertensive heart disease mortality rates, 2012-2015; (B) average annual percent change of hypertensive heart disease mortality rates, 2000-2015, and (C) multivariate quasi-Poisson regression by county-level risk factors 2012-15
eTable 1. Trends and age-standardized premature mortality (age 25-64 years) rates due to specific cardiovascular disease in the US, 2000-03 compared to 2012-15, rates per 100,000
eTable 2. Relative risk and 95% confidence intervals for county-level CVD premature mortality by country-level risk factors, adjusting for age and all five county-level risk factors, 2012-15
eTable 3. Relative risk and 95% confidence intervals (CI) for county-level premature mortality due to hypertensive heart disease by county-level risk factors, adjusting for age and all five county-level risk factors, 2012-15

This supplementary materials has been provided by the authors to give readers additional information about their work.

© 2019 Chen et al. JAMA Cardiology.

eFigure 1. Average annual percent change of CVD mortality rates by States, stratified by race/ethnicity, sex, and age groups, 2000–15

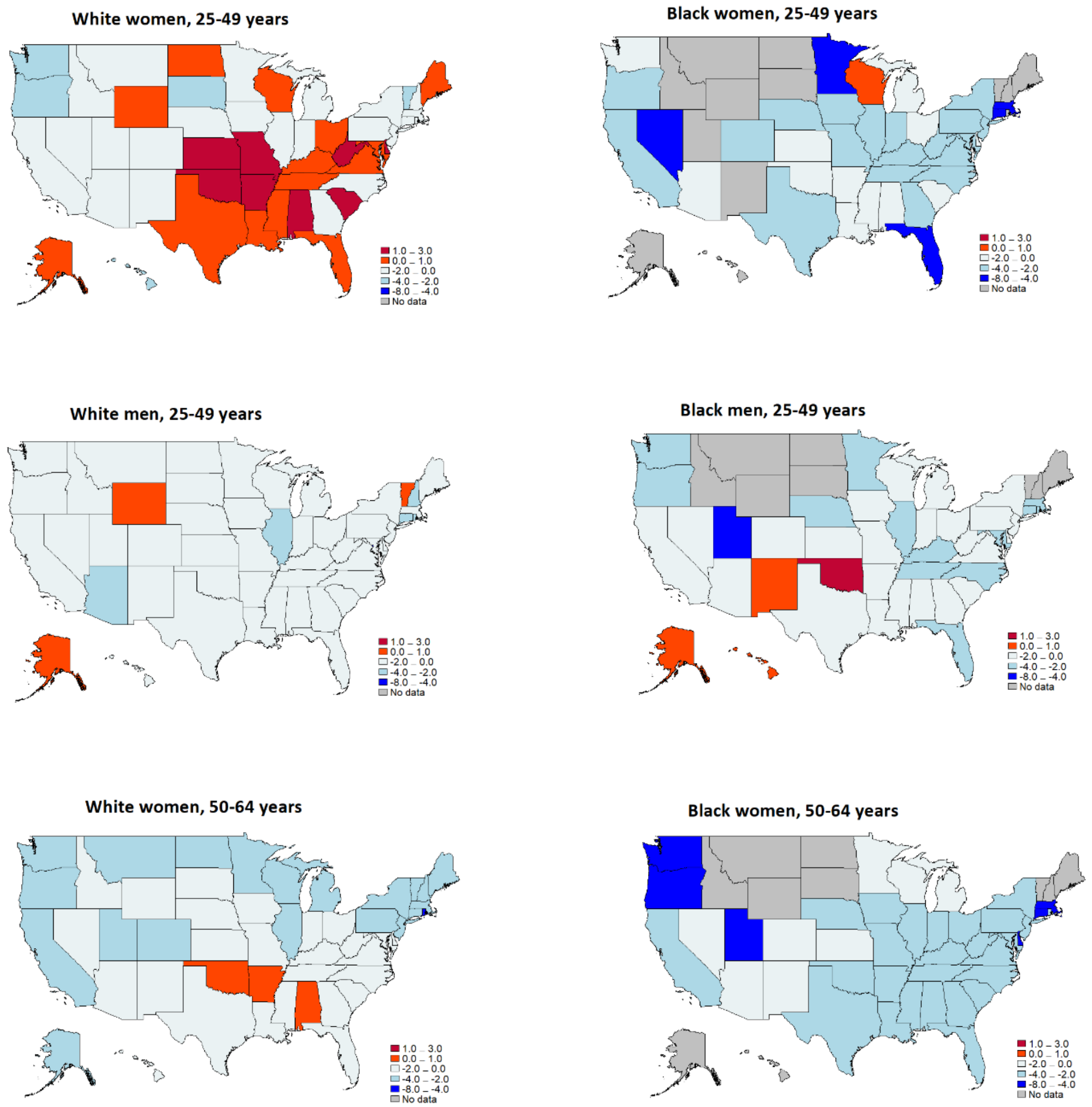

© 2019 Chen et al. JAMA Cardiology.

eFigure 2. Average annual percent change of mortality rates due to hypertensive heart disease by States, 2000–15

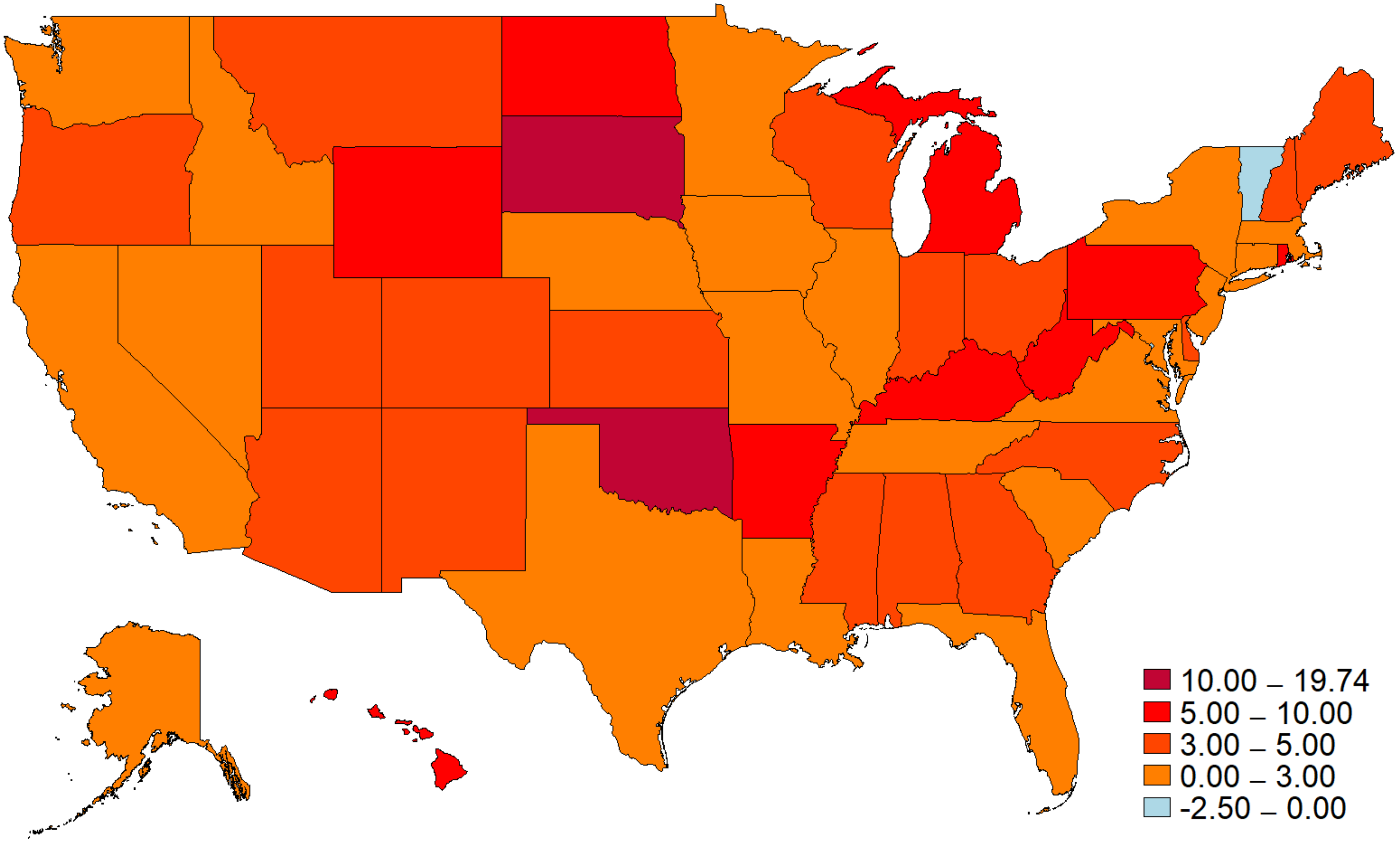

© 2019 Chen et al. JAMA Cardiology.

eFigure 3. County-level A) age-standardized hypertensive heart disease mortality rates, 2012–2015; B) average annual percent change of hypertensive heart disease mortality rates, 2000–2015, and C) Multivariate Quasi-Poisson regression by county-level risk factors 2012-15.

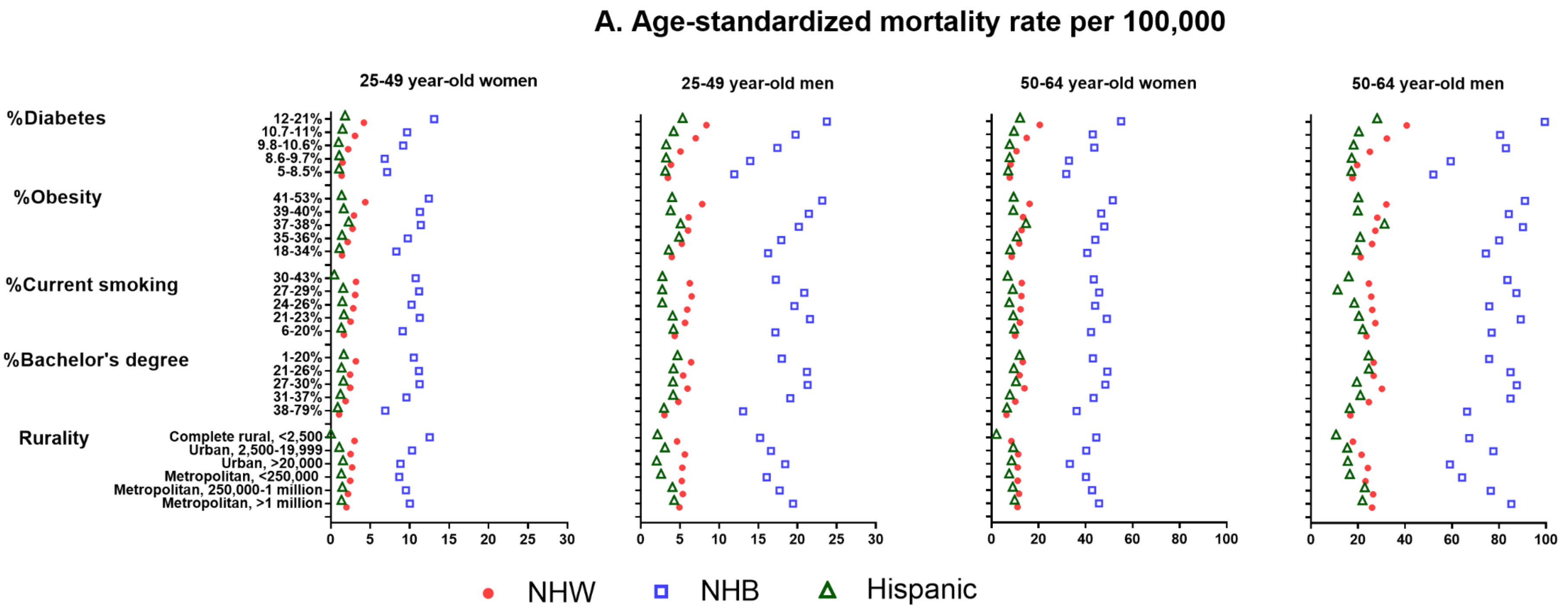

© 2019 Chen et al. JAMA Cardiology.

B. Average annual percent change, 2000-15

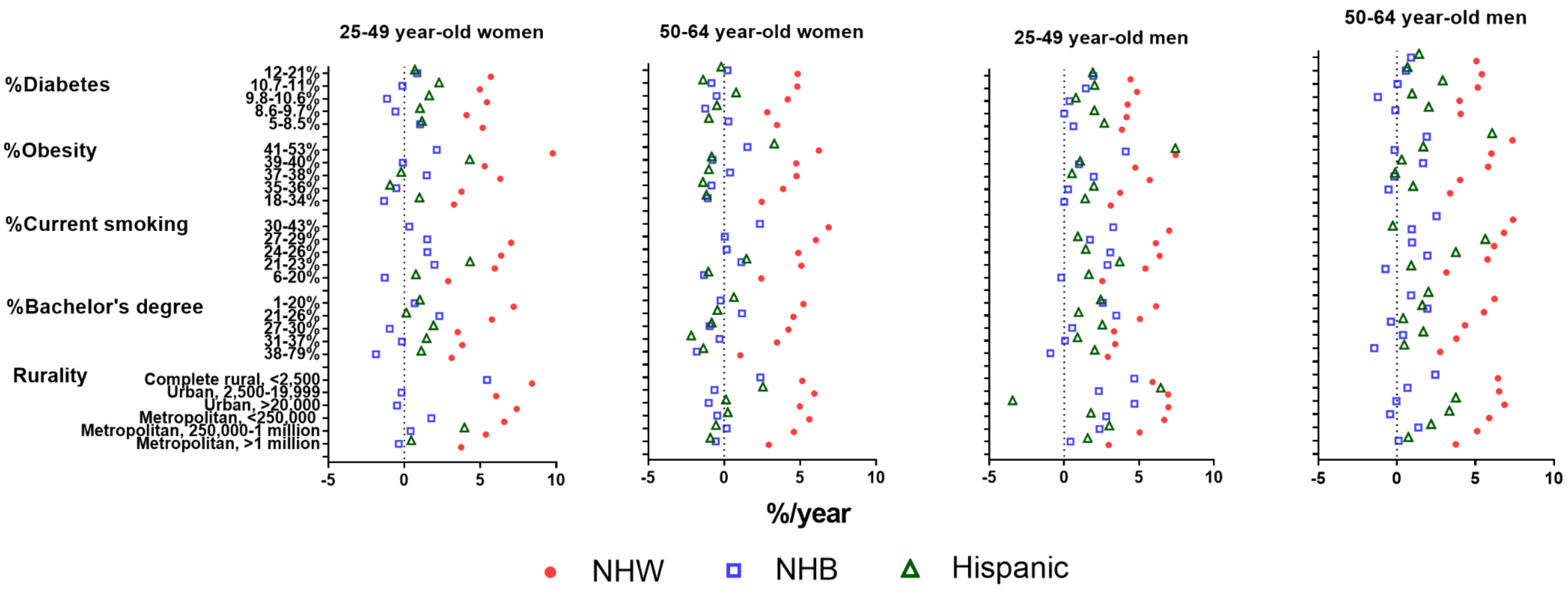

© 2019 Chen et al. JAMA Cardiology.

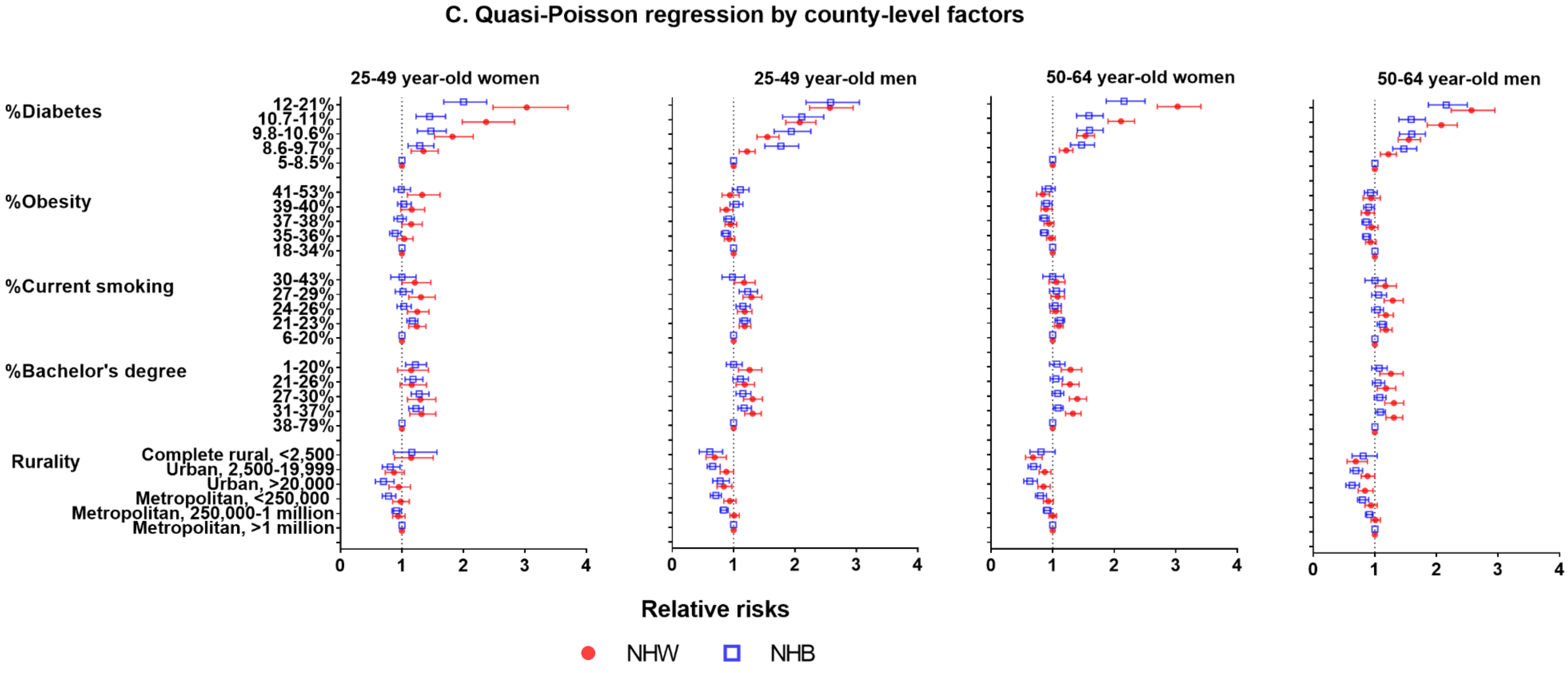

© 2019 Chen et al. JAMA Cardiology.

eTable 1. Trends and age-standardized premature mortality (age 25-64 years) rates due to specific cardiovascular disease in the US, 2000–03 compared to 2012–15, rates per 100,000.

Ischemic heart disease

Women									
Race/ethnicity	25-49-year-old			50-64-year-old			overall		
	ASR/100000, 2000–2003	ASR/100000, 2012–2015	average annual percent change	ASR/100000, 2000–2003	ASR/100000, 2012–2015	average annual percent change	ASR/100000, 2000–2003	ASR/100000, 2012–2015	average annual percent change
White	7.33	6.95	-0.488	68.30	48.39	-2.891	24.90	18.89	-2.356
Black	17.72	11.70	-3.512	153.81	87.11	-4.707	56.95	33.44	-4.437
Latino	3.78	1.58	-2.334	54.04	31.08	-4.579	18.27	11.00	-4.206
API	2.07	1.58	-2.037	29.15	16.96	-4.231	9.87	6.01	-3.866
AI/AN	9.93	12.24	1.741	93.41	78.29	-1.495	33.99	31.28	-0.747
Overall	8.04	6.58	-1.72	75.17	49.88	-3.42	27.39	19.06	-3.052
Men									
Race/ethnicity	25-49-year-old			50-64-year-old			overall		
	ASR/100000, 2000–2003	ASR/100000, 2012–2015	average annual percent change	ASR/100000, 2000–2003	ASR/100000, 2012–2015	average annual percent change	ASR/100000, 2000–2003	ASR/100000, 2012–2015	average annual percent change
White	27.71	20.28	-2.507	206.93	146.94	-2.85	79.37	56.79	-2.791
Black	39.05	28.39	-2.74	322.19	199.41	-4.033	120.66	77.69	-3.723
Latino	14.00	10.30	-2.62	151.16	97.43	-3.641	53.53	35.42	-3.438
API	13.14	10.92	-1.491	100.16	72.06	-2.739	38.22	28.54	-2.414
AI/AN	35.25	35.63	0.13	251.92	219.67	-0.984	97.71	88.68	-0.674
Overall	26.96	19.06	-2.843	210.74	144.87	-3.118	79.94	55.32	-3.068

© 2019 Chen et al. JAMA Cardiology.

Cerebrovascular disease

Women									
Race/ethnicity	25-49-year-old			50-64-year-old			overall		
	ASR/100000, 2000–2003	ASR/100000, 2012–2015	average annual percent change	ASR/100000, 2000–2003	ASR/100000, 2012–2015	average annual percent change	ASR/100000, 2000–2003	ASR/100000, 2012–2015	average annual percent change
White	3.82	3.06	-1.877	21.11	16.20	-2.204	8.80	6.84	-2.136
Black	13.39	7.62	-4.481	61.95	41.06	-3.39	27.38	17.26	-3.779
Latino	4.30	3.01	-3.133	23.18	15.59	-3.34	9.75	6.63	-3.264
API	3.94	2.92	-2.151	25.71	15.61	-4.047	10.21	6.58	-3.463
AI/AN	9.92	6.70	-2.814	34.83	26.11	-1.89	17.10	12.30	-2.251
Overall	5.12	3.69	-2.702	25.94	19.20	-2.488	11.12	8.16	-2.579
Men									
Race/ethnicity	25-49-year-old			50-64-year-old			overall		
	ASR/100000, 2000–2003	ASR/100000, 2012–2015	average annual percent change	ASR/100000, 2000–2003	ASR/100000, 2012–2015	average annual percent change	ASR/100000, 2000–2003	ASR/100000, 2012–2015	average annual percent change
White	4.17	3.53	-1.399	26.72	22.28	-1.543	10.67	8.94	-1.527
Black	14.50	10.80	-2.576	92.17	63.45	-3.133	36.88	25.97	-2.962
Latino	6.04	4.67	-2.321	36.01	26.54	-2.666	14.68	10.98	-2.57
API	5.44	4.05	-1.878	33.61	24.55	-2.583	13.56	9.96	-2.395
AI/AN	7.88	6.65	-0.419	38.71	35.93	-1.417	16.77	15.09	-1.033
Overall	5.65	4.70	-1.569	33.99	27.50	-1.797	13.82	11.27	-1.742

Hypertensive heart disease

© 2019 Chen et al. JAMA Cardiology.

Women									
Race/ethnicity	25-49-year-old			50-64-year-old			overall		
	ASR/100000, 2000–2003	ASR/100000, 2012–2015	average annual percent change	ASR/100000, 2000–2003	ASR/100000, 2012–2015	average annual percent change	ASR/100000, 2000–2003	ASR/100000, 2012–2015	average annual percent change
White	1.22	2.20	5.05	7.11	11.10	3.976	2.91	4.77	4.292
Black	9.94	9.89	-0.002	46.88	44.25	-0.382	20.59	19.79	-0.272
Latino	1.18	1.37	1.337	9.68	9.39	-0.683	3.63	3.68	-0.198
API	0.63	0.58	-0.493	5.06	4.56	-1.335	1.91	1.72	-1.123
AI/AN	1.90	5.48	9.078	7.56	18.60	5.203	3.54	9.26	6.802
Overall	2.31	3.02	2.265	11.53	14.71	2.153	4.97	6.39	2.168
Men									
Race/ethnicity	25-49-year-old			50-64-year-old			overall		
	ASR/100000, 2000–2003	ASR/100000, 2012–2015	average annual percent change	ASR/100000, 2000–2003	ASR/100000, 2012–2015	average annual percent change	ASR/100000, 2000–2003	ASR/100000, 2012–2015	average annual percent change
White	3.04	5.11	4.34	14.18	24.90	4.694	6.25	10.81	4.539
Black	16.25	18.64	1.107	76.10	80.48	0.316	33.50	36.47	0.597
Latino	3.28	4.03	1.846	18.52	21.31	1.281	7.68	9.01	1.457
API	1.91	2.50	1.922	9.69	12.84	2.232	4.15	5.48	2.16
AI/AN	3.00	11.71	8.975	18.85	46.61	8.158	7.57	21.77	8.613
Overall	4.55	6.49	2.933	20.32	30.31	3.295	9.10	13.35	3.149

Heart failure

Women

© 2019 Chen et al. JAMA Cardiology.

Race/ethnicity	25-49-year-old			50-64-year-old			overall		
	ASR/100000, 2000–2003	ASR/100000, 2012–2015	average annual percent change	ASR/100000, 2000–2003	ASR/100000, 2012–2015	average annual percent change	ASR/100000, 2000–2003	ASR/100000, 2012–2015	average annual percent change
White	1.72	1.62	-0.317	9.85	7.97	-1.838	4.07	3.45	-1.398
Black	7.42	5.39	-2.712	29.56	23.37	-2.032	13.80	10.58	-2.29
Latino	1.17	0.89	-1.868	7.54	5.60	-2.169	3.00	2.25	-2.096
API	0.76	0.61	-2.257	4.70	3.53	-2.628	1.90	1.45	-2.395
AI/AN	2.74	4.77	3.754	14.57	10.01	-3.299	6.15	6.28	-0.473
Overall	2.36	1.96	-1.461	11.63	9.39	-1.84	5.03	4.10	-1.742
Men									
Race/ethnicity	25-49-year-old			50-64-year-old			overall		
	ASR/100000, 2000–2003	ASR/100000, 2012–2015	average annual percent change	ASR/100000, 2000–2003	ASR/100000, 2012–2015	average annual percent change	ASR/100000, 2000–2003	ASR/100000, 2012–2015	average annual percent change
White	4.28	3.71	-1.162	19.51	16.92	-1.118	8.67	7.52	-1.18
Black	14.72	11.52	-2.157	54.91	44.90	-1.768	26.31	21.14	-1.943
Latino	3.18	2.63	-1.598	15.18	12.02	-1.77	6.64	5.33	-1.737
API	2.03	2.31	0.63	8.64	8.18	-0.399	3.93	4.00	-0.025
AI/AN	9.28	9.37	0.239	28.83	25.36	-0.803	14.91	13.98	-0.31
Overall	5.31	4.43	-1.531	22.24	19.15	-1.2	10.19	8.67	-1.358

Peripheral arterial disease

Women

© 2019 Chen et al. JAMA Cardiology.

Race/ethnicity	25-49-year-old			50-64-year-old			overall		
	ASR/100000, 2000–2003	ASR/100000, 2012–2015	average annual percent change	ASR/100000, 2000–2003	ASR/100000, 2012–2015	average annual percent change	ASR/100000, 2000–2003	ASR/100000, 2012–2015	average annual percent change
White	0.64	0.56	-1.27	4.67	3.12	-3.378	1.80	1.30	-2.821
Black	1.73	1.16	-3.398	9.29	5.62	-4.251	3.91	2.44	-3.975
Latino	0.53	0.39	-3.033	3.28	1.71	-5.487	1.32	0.77	-4.696
API	0.40	0.32	-1.344	2.22	1.67	-2.585	0.92	0.71	-2.115
AI/AN	0.87	1.54		8.86	5.95	-4.366	3.17	2.81	-1.874
Overall	0.76	0.60	-2.078	4.99	3.23	-3.655	1.98	1.36	-3.209
Men									
Race/ethnicity	25-49-year-old			50-64-year-old			overall		
	ASR/100000, 2000–2003	ASR/100000, 2012–2015	average annual percent change	ASR/100000, 2000–2003	ASR/100000, 2012–2015	average annual percent change	ASR/100000, 2000–2003	ASR/100000, 2012–2015	average annual percent change
White	1.39	1.31	-0.541	10.44	7.08	-3.216	4.00	2.97	-2.498
Black	3.23	3.11	-0.533	17.67	11.14	-3.971	7.39	5.43	-2.763
Latino	0.97	0.96	-0.089	6.61	3.95	-4.308	2.60	1.83	-2.976
API	0.85	0.80	-0.68	5.75	4.12	-3.23	2.26	1.76	-2.471
AI/AN	1.16	1.27	1.314	10.76	6.86	-3.585	3.93	2.88	-2.596
Overall	1.53	1.44	-0.632	10.72	7.10	-3.445	4.18	3.07	-2.631

Cardiac arrest

Women

© 2019 Chen et al. JAMA Cardiology.

Race/ethnicity	25-49-year-old			50-64-year-old			overall		
	ASR/100000, 2000–2003	ASR/100000, 2012–2015	average annual percent change	ASR/100000, 2000–2003	ASR/100000, 2012–2015	average annual percent change	ASR/100000, 2000–2003	ASR/100000, 2012–2015	average annual percent change
White	0.51	0.65	2.012	3.44	3.39	0.151	1.35	1.44	0.687
Black	1.63	1.47	-1.126	8.79	7.38	-1.346	3.70	3.18	-1.279
Latino	0.20	0.23	1.754	1.16	1.32	2.097	0.48	0.55	2.002
API	0.26	0.14	-5.105	1.61	1.36	-2.621	0.65	0.49	-2.956
AI/AN	0.48	0.68	NA	4.87	2.14	-4.186	1.75	1.10	-3.341
Overall	0.61	0.65	0.522	3.78	3.54	-0.282	1.52	1.49	-0.054
Men									
Race/ethnicity	25-49-year-old			50-64-year-old			overall		
	ASR/100000, 2000–2003	ASR/100000, 2012–2015	average annual percent change	ASR/100000, 2000–2003	ASR/100000, 2012–2015	average annual percent change	ASR/100000, 2000–2003	ASR/100000, 2012–2015	average annual percent change
White	1.22	1.03	-1.262	7.73	6.43	-1.44	3.10	2.59	-1.42
Black	2.61	2.23	-1.431	16.74	13.31	-1.686	6.69	5.42	-1.622
Latino	0.39	0.41	0.839	2.28	2.65	1.75	0.94	1.06	1.472
API	0.54	0.46	-0.86	4.12	2.76	-3.347	1.57	1.12	-2.63
AI/AN	1.47	1.19	-0.285	11.51	6.01	-3.065	4.37	2.58	-2.838
Overall	1.26	1.03	-1.603	8.07	6.60	-1.521	3.22	2.63	-1.564

Arrhythmia

Women

© 2019 Chen et al. JAMA Cardiology.

Race/ethnicity	25-49-year-old			50-64-year-old			overall		
	ASR/100000, 2000–2003	ASR/100000, 2012–2015	average annual percent change	ASR/100000, 2000–2003	ASR/100000, 2012–2015	average annual percent change	ASR/100000, 2000–2003	ASR/100000, 2012–2015	average annual percent change
White	0.68	0.71	0.538	2.35	2.27	-0.233	1.16	1.16	0.075
Black	1.47	1.30	-1.392	5.04	4.43	-1.197	2.50	2.20	-1.313
Latino	0.21	0.19	-0.464	1.10	0.97	-0.866	0.46	0.41	-0.797
API	0.17	0.14	-1.254	0.50	0.66	1.126	0.27	0.29	0.398
AI/AN	0.85	0.94	2.011	3.77	2.49	-3.915	1.69	1.38	-1.051
Overall	0.71	0.66	-0.529	2.48	2.32	-0.526	1.22	1.14	-0.549
Men									
Race/ethnicity	25-49-year-old			50-64-year-old			overall		
	ASR/100000, 2000–2003	ASR/100000, 2012–2015	average annual percent change	ASR/100000, 2000–2003	ASR/100000, 2012–2015	average annual percent change	ASR/100000, 2000–2003	ASR/100000, 2012–2015	average annual percent change
White	1.01	0.94	-0.648	4.28	4.45	0.385	1.95	1.95	-0.013
Black	1.73	1.73	-0.09	8.72	6.99	-1.771	3.74	3.25	-1.197
Latino	0.46	0.36	-2.114	1.74	1.69	-0.241	0.83	0.74	-0.951
API	0.40	0.35	-1.89	1.12	1.30	-0.16	0.61	0.62	-0.752
AI/AN	1.14	1.24	1.197	4.55	5.87	1.996	2.12	2.58	1.524
Overall	1.01	0.90	-1.023	4.41	4.29	-0.167	1.99	1.88	-0.499

Endocarditis

Women

© 2019 Chen et al. JAMA Cardiology.

Race/ethnicity	25-49-year-old			50-64-year-old			overall		
	ASR/100000, 2000–2003	ASR/100000, 2012–2015	average annual percent change	ASR/100000, 2000–2003	ASR/100000, 2012–2015	average annual percent change	ASR/100000, 2000–2003	ASR/100000, 2012–2015	average annual percent change
White	0.31	0.46	3.835	1.24	1.13	-0.767	0.57	0.65	1.163
Black	1.09	0.55	-5.11	3.72	2.43	-3.371	1.85	1.09	-4.064
Latino	0.23	0.18	-1.583	1.32	0.91	-2.793	0.55	0.39	-2.402
API	0.16	0.12	NA	0.72	0.51	-3.417	0.32	0.23	-2.925
AI/AN	0.86	1.59	NA	2.12	1.48	-5.244	1.23	1.56	-1.812
Overall	0.40	0.40	0.423	1.50	1.24	-1.589	0.71	0.64	-0.773

Men

Race/ethnicity	25-49-year-old			50-64-year-old			overall		
	ASR/100000, 2000–2003	ASR/100000, 2012–2015	average annual percent change	ASR/100000, 2000–2003	ASR/100000, 2012–2015	average annual percent change	ASR/100000, 2000–2003	ASR/100000, 2012–2015	average annual percent change
White	0.49	0.79	3.981	1.71	1.95	0.828	0.84	1.12	2.25
Black	1.57	0.87	-4.957	5.38	3.91	-3.098	2.67	1.75	-3.747
Latino	0.49	0.44	-2.388	1.77	1.97	0.026	0.86	0.88	-0.947
API	0.18	0.22	0.993	0.74	0.82	0.539	0.34	0.39	0.799
AI/AN	1.48	2.77	5.606	1.50	3.80	NA	1.48	3.06	3.902
Overall	0.61	0.70	0.916	2.04	2.13	-0.023	1.02	1.11	0.41

Rheumatic heart disease

Women

© 2019 Chen et al. JAMA Cardiology.

Race/ethnicity	25-49-year-old			50-64-year-old			overall		
	ASR/100000, 2000–2003	ASR/100000, 2012–2015	average annual percent change	ASR/100000, 2000–2003	ASR/100000, 2012–2015	average annual percent change	ASR/100000, 2000–2003	ASR/100000, 2012–2015	average annual percent change
White	0.21	0.14	-3.203	1.27	0.63	-5.765	0.52	0.28	-4.973
Black	0.61	0.28	-6.592	2.27	1.21	-4.288	1.09	0.55	-5.159
Latino	0.29	0.08	-10.613	1.33	0.72	-5.347	0.59	0.26	-6.723
API	0.35	0.17	-4.747	1.65	0.96	-4.891	0.72	0.40	-4.952
AI/AN	0.49	0.29	NA	3.22	1.45	NA	1.27	0.63	-3.029
Overall	0.28	0.15	-4.87	1.41	0.73	-5.241	0.60	0.32	-5.109
Men									
Race/ethnicity	25-49-year-old			50-64-year-old			overall		
	ASR/100000, 2000–2003	ASR/100000, 2012–2015	average annual percent change	ASR/100000, 2000–2003	ASR/100000, 2012–2015	average annual percent change	ASR/100000, 2000–2003	ASR/100000, 2012–2015	average annual percent change
White	0.18	0.14	-2.817	0.86	0.56	-3.558	0.38	0.26	-3.333
Black	0.42	0.28	-2.911	1.46	0.90	-3.349	0.72	0.46	-3.058
Latino	0.17	0.12	-1.808	0.89	0.54	-4.071	0.38	0.24	NA
API	0.21	0.15	-2.259	0.64	0.66	-0.344	0.34	0.29	-1.012
AI/AN	0.41	0.08	NA	1.22	1.78	NA	0.64	0.57	-3.235
Overall	0.21	0.16	-2.727	0.92	0.61	-3.272	0.42	0.29	-3.07

© 2019 Chen et al. JAMA Cardiology.

eTable 2. Relative risk and 95% confidence intervals for county-level CVD premature mortality by county-level risk factors, adjusting for age and all five county-level risk factors, 2012–15.

County-level risk factors	25-49-year-old women						25-49-year-old men						50-64-year-old women						50-64-year-old men					
	NHW			NHB			NHW			NHB			NHW			NHB			NHW			NHB		
**% diabetes**	RR	lci	uci	RR	lci	uci	RR	lci	uci	RR	lci	uci	RR	lci	uci	RR	lci	uci	RR	lci	uci	RR	lci	uci
12-21%	1.82	1.70	1.95	1.39	1.26	1.54	1.61	1.54	1.70	1.60	1.47	1.75	1.60	1.47	1.75	1.37	1.28	1.47	1.62	1.58	1.67	1.55	1.47	1.64
10.7-11%	1.59	1.50	1.69	1.25	1.14	1.37	1.48	1.42	1.54	1.45	1.34	1.57	1.45	1.34	1.57	1.24	1.16	1.32	1.48	1.45	1.52	1.37	1.30	1.44
9.8-10.6%	1.36	1.28	1.44	1.27	1.16	1.39	1.29	1.24	1.34	1.37	1.27	1.48	1.37	1.27	1.48	1.23	1.16	1.31	1.31	1.28	1.34	1.36	1.29	1.43
8.6-9.7%	1.12	1.06	1.18	1.14	1.04	1.25	1.12	1.08	1.16	1.18	1.09	1.27	1.18	1.09	1.27	1.13	1.06	1.20	1.15	1.12	1.17	1.19	1.13	1.25
5-8.5%	reference			reference			reference			reference			reference			reference			reference			reference		
**% obesity**																								
41-53%	1.04	0.97	1.12	1.25	1.15	1.36	1.02	0.96	1.07	1.21	1.12	1.30	1.21	1.12	1.30	1.16	1.09	1.22	0.96	0.93	0.99	1.13	1.08	1.19
39-40%	1.10	1.04	1.17	1.19	1.12	1.27	1.00	0.95	1.04	1.09	1.03	1.16	1.09	1.03	1.16	1.09	1.04	1.14	0.97	0.95	1.00	1.05	1.01	1.09
37-38%	1.05	1.00	1.11	1.12	1.05	1.19	0.99	0.96	1.03	1.01	0.95	1.06	1.01	0.95	1.06	1.04	1.00	1.09	0.96	0.94	0.98	1.02	0.98	1.05
35-36%	1.04	0.99	1.09	1.01	0.95	1.07	0.97	0.94	1.00	0.97	0.92	1.02	0.97	0.92	1.02	1.02	0.98	1.06	0.95	0.93	0.97	0.98	0.95	1.01
18-34%	reference			reference			reference			reference			reference			reference			reference			reference		
**% current smoking**																								
30-43%	1.44	1.35	1.53	1.22	1.10	1.36	1.36	1.30	1.43	1.09	0.99	1.21	1.09	0.99	1.21	1.21	1.13	1.30	1.20	1.17	1.24	1.32	1.24	1.39
27-29%	1.31	1.23	1.39	1.15	1.06	1.24	1.24	1.19	1.30	1.15	1.07	1.24	1.15	1.07	1.24	1.08	1.02	1.14	1.14	1.11	1.17	1.17	1.12	1.22
24-26%	1.28	1.22	1.35	1.19	1.11	1.26	1.20	1.16	1.25	1.24	1.17	1.31	1.24	1.17	1.31	1.12	1.07	1.17	1.13	1.11	1.15	1.16	1.12	1.20
21-23%	1.20	1.15	1.26	1.16	1.11	1.22	1.16	1.13	1.20	1.19	1.14	1.25	1.19	1.14	1.25	1.15	1.11	1.19	1.12	1.10	1.14	1.17	1.14	1.21
6-20%	reference			reference			reference			reference			reference			reference			reference			reference		
**% bachelor’s degree**																								
1-20%	1.48	1.37	1.60	0.97	0.90	1.06	1.35	1.28	1.42	0.90	0.83	0.97	0.90	0.83	0.97	1.04	0.98	1.10	1.36	1.32	1.40	0.95	0.90	0.99
21-26%	1.32	1.24	1.41	1.04	0.97	1.12	1.26	1.20	1.31	0.98	0.92	1.05	0.98	0.92	1.05	1.04	0.99	1.09	1.25	1.22	1.29	0.97	0.93	1.01
27-30%	1.30	1.22	1.39	1.14	1.07	1.22	1.26	1.21	1.32	1.07	1.01	1.13	1.07	1.01	1.13	1.09	1.05	1.15	1.23	1.20	1.26	1.06	1.02	1.10
31-37%	1.21	1.14	1.28	1.07	1.01	1.14	1.15	1.11	1.20	1.03	0.98	1.09	1.03	0.98	1.09	1.01	0.97	1.05	1.15	1.13	1.18	1.01	0.98	1.05
38-79%	reference			reference			reference			reference			reference			reference			reference			reference		
**rurality**																								
completely rural, <2,500	1.28	1.17	1.41	1.38	1.16	1.64	1.22	1.14	1.30	0.84	0.72	0.99	0.84	0.72	0.99	0.88	0.78	1.00	1.04	1.00	1.08	0.94	0.86	1.03
urban, 2,500 to <20,000	1.26	1.19	1.33	1.42	1.30	1.56	1.17	1.12	1.22	1.01	0.92	1.09	1.01	0.92	1.09	1.02	0.95	1.08	1.05	1.03	1.08	0.99	0.95	1.05
urban, ≥20,000	1.18	1.11	1.26	1.10	0.98	1.23	1.11	1.06	1.16	1.00	0.91	1.10	1.00	0.91	1.10	0.92	0.85	0.99	1.00	0.98	1.03	0.89	0.84	0.95
Metropolitan, <250,000	1.17	1.11	1.23	1.16	1.08	1.25	1.14	1.10	1.18	0.98	0.92	1.05	0.98	0.92	1.05	1.03	0.98	1.08	1.01	0.99	1.04	0.96	0.92	1.00
Metropolitan, 250,000 to < 1m	1.15	1.11	1.20	1.14	1.09	1.19	1.10	1.08	1.13	1.05	1.01	1.10	1.05	1.01	1.10	1.04	1.01	1.08	1.01	1.00	1.03	0.99	0.96	1.01
Metropolitan, ≥1m	reference			reference			reference			reference			reference			reference			reference			reference		

© 2019 Chen et al. JAMA Cardiology.

eTable 3. Relative risk and 95% confidence intervals (CI) for county-level premature mortality due to hypertensive heart disease by county-level risk factors, adjusting for age and all five county-level risk factors, 2012–15.

County-level risk factors	25-49-year-old women						25-49-year-old men						50-64-year-old women						50-64-year-old men					
	NHW			NHB			NHW			NHB			NHW			NHB			NHW			NHB		
**% diabetes**	RR	lci	uci	RR	lci	uci	RR	lci	uci	RR	lci	uci	RR	lci	uci	RR	lci	uci	RR	lci	uci	RR	lci	uci
12-21%	3.03	2.48	3.70	2.00	1.68	2.38	2.57	2.24	2.95	2.58	2.18	3.05	3.03	2.70	3.41	2.16	1.87	2.50	2.57	2.24	2.95	2.16	1.87	2.50
10.7-11%	2.37	1.98	2.83	1.45	1.23	1.71	2.08	1.85	2.34	2.11	1.80	2.47	2.11	1.90	2.33	1.59	1.39	1.82	2.08	1.85	2.34	1.59	1.39	1.82
9.8-10.6%	1.82	1.53	2.16	1.47	1.25	1.72	1.55	1.38	1.74	1.94	1.66	2.26	1.53	1.39	1.68	1.60	1.40	1.82	1.55	1.38	1.74	1.60	1.40	1.82
8.6-9.7%	1.35	1.15	1.59	1.29	1.10	1.52	1.22	1.09	1.35	1.77	1.51	2.06	1.22	1.11	1.33	1.47	1.29	1.68	1.22	1.09	1.35	1.47	1.29	1.68
5-8.5%	reference			reference			reference			reference			reference			reference			reference			reference		
**% obesity**																								
41-53%	1.33	1.09	1.62	0.99	0.87	1.14	0.94	0.81	1.09	1.11	0.98	1.25	0.84	0.74	0.95	0.93	0.83	1.04	0.94	0.81	1.09	0.93	0.83	1.04
39-40%	1.16	0.98	1.37	1.03	0.93	1.15	0.88	0.78	0.99	1.04	0.94	1.15	0.89	0.81	0.99	0.90	0.82	0.99	0.88	0.78	0.99	0.90	0.82	0.99
37-38%	1.15	1.00	1.33	0.97	0.87	1.07	0.95	0.86	1.05	0.92	0.84	1.01	0.94	0.86	1.02	0.86	0.79	0.94	0.95	0.86	1.05	0.86	0.79	0.94
35-36%	1.04	0.92	1.18	0.89	0.80	0.98	0.93	0.85	1.02	0.87	0.80	0.95	0.97	0.90	1.04	0.86	0.80	0.93	0.93	0.85	1.02	0.86	0.80	0.93
18-34%	reference			reference			reference			reference			reference			reference			reference			reference		
**% current smoking**																								
30-43%	1.21	1.00	1.47	1.00	0.82	1.23	1.17	1.01	1.35	0.98	0.81	1.18	1.06	0.94	1.20	1.00	0.84	1.18	1.17	1.01	1.35	1.00	0.84	1.18
27-29%	1.31	1.11	1.54	1.02	0.89	1.17	1.29	1.15	1.46	1.23	1.09	1.39	1.08	0.97	1.19	1.06	0.95	1.19	1.29	1.15	1.46	1.06	0.95	1.19
24-26%	1.25	1.09	1.44	1.03	0.92	1.15	1.18	1.06	1.30	1.15	1.04	1.27	1.05	0.96	1.14	1.04	0.95	1.14	1.18	1.06	1.30	1.04	0.95	1.14
21-23%	1.24	1.11	1.39	1.17	1.08	1.26	1.18	1.09	1.28	1.18	1.10	1.27	1.10	1.03	1.17	1.12	1.04	1.19	1.18	1.09	1.28	1.12	1.04	1.19
6-20%	reference			reference			reference			reference			reference			reference			reference			reference		
**% bachelor’s degree**																								
1-20%	1.15	0.93	1.43	1.22	1.06	1.40	1.26	1.08	1.46	1.00	0.88	1.14	1.29	1.14	1.47	1.07	0.95	1.20	1.26	1.08	1.46	1.07	0.95	1.20
21-26%	1.16	0.97	1.40	1.18	1.05	1.34	1.18	1.04	1.34	1.11	0.99	1.24	1.28	1.15	1.43	1.05	0.96	1.16	1.18	1.04	1.34	1.05	0.96	1.16
27-30%	1.30	1.09	1.55	1.28	1.15	1.44	1.31	1.16	1.47	1.15	1.04	1.28	1.40	1.27	1.55	1.08	0.99	1.18	1.31	1.16	1.47	1.08	0.99	1.18
31-37%	1.32	1.13	1.55	1.23	1.11	1.35	1.31	1.18	1.45	1.17	1.07	1.29	1.33	1.21	1.46	1.09	1.01	1.17	1.31	1.18	1.45	1.09	1.01	1.17
38-79%	reference			reference			reference			reference			reference			reference			reference			reference		
**rurality**																								
completely rural, <2,500	1.15	0.88	1.51	1.16	0.86	1.57	0.69	0.55	0.88	0.61	0.44	0.82	0.68	0.56	0.83	0.81	0.63	1.04	0.69	0.55	0.88	0.81	0.63	1.04
urban, 2,500 to <20,000	0.87	0.73	1.04	0.81	0.68	0.97	0.88	0.78	1.00	0.66	0.57	0.78	0.87	0.78	0.97	0.69	0.60	0.80	0.88	0.78	1.00	0.69	0.60	0.80
urban, ≥20,000	0.95	0.79	1.14	0.70	0.57	0.87	0.84	0.73	0.97	0.78	0.66	0.93	0.85	0.76	0.96	0.63	0.53	0.75	0.84	0.73	0.97	0.63	0.53	0.75
Metropolitan, <250,000	0.98	0.85	1.12	0.78	0.68	0.90	0.94	0.84	1.04	0.71	0.62	0.80	0.93	0.85	1.01	0.80	0.72	0.90	0.94	0.84	1.04	0.80	0.72	0.90
Metropolitan, 250,000 to < 1m	0.94	0.85	1.05	0.91	0.84	0.99	1.01	0.94	1.09	0.84	0.78	0.91	1.00	0.94	1.06	0.91	0.85	0.97	1.01	0.94	1.09	0.91	0.85	0.97
Metropolitan, ≥1m	reference			reference			reference			reference			reference			reference			reference			reference		

© 2019 Chen et al. JAMA Cardiology.
